# Hydrophobic Flocculation of Fine Cassiterite Using Alkyl Hydroxamic Acids with Different Carbon Chain Lengths as Collectors

**DOI:** 10.3390/molecules28093911

**Published:** 2023-05-05

**Authors:** Saizhen Jin, Qing Shi, Leming Ou

**Affiliations:** School of Minerals Processing and Bioengineering, Central South University, Changsha 410083, China; jinsaizhen@csu.edu.cn (S.J.);

**Keywords:** fine cassiterite, alkyl hydroxamates, hydrophobic flocculation, carbon chain length

## Abstract

This work investigated the hydrophobic flocculation of cassiterite using four alkyl hydroxamic acids with varying carbon chain lengths, i.e., hexyl hydroxamate (C_6_), octyl hydroxamate (C_8_), decyl hydroxamate (C_10_) and dodecyl hydroxamate (C_12_), as collectors. Microflotation tests were performed to investigate the flotation behaviour of cassiterite in the presence of the four alkyl hydroxamic acids. Focused beam reflectance measurement (FBRM) and a particle video microscope (PVM) were used to analyse and monitor the real-time evolution of the particle size distribution of cassiterite and the images of flocs during flocculation. The extended DLVO theory interaction energies between the cassiterite particles were calculated on the basis of the measured contact angle and the zeta potential of cassiterite to determine the aggregation and dispersion behaviour of the cassiterite particles. The microflotation test results suggested that the floatability of cassiterite improved with the increase in the carbon chain length of hydroxamates. FBRM, PVM images and extended DLVO theory calculation results indicated that when C_6_ was used as the collector, the cassiterite particles could not form hydrophobic flocs because the total potential energy between them was repulsive. When C_8_, C_10_ and C_12_ were used as collectors, the energy barrier amongst particles decreased with increasing hydroxamate concentration. The lowest concentrations of C_8_, C_10_ and C_12_ that could cause the hydrophobic aggregation of cassiterite were approximately 1 × 10^−3^, 1 × 10^−4^ and 2 × 10^−5^ mol/L, respectively. The aggregation growth rate and apparent floc size increased with an increasing collector concentration. Hydroxamic acid with a longer carbon chain could induce the cassiterite particles to form larger flocs at a lower concentration in a shorter time.

## 1. Introduction

Many primary tin ores intergrow with other minerals in the form of fine particles [1]. The brittleness of cassiterite [2] results in the generation of a considerable volume of fine particles during crushing and grinding. Froth flotation is the most effective method for separating fine-grained minerals [3,4,5]. However, fine particles have low probabilities of collision with bubbles due to their intrinsic physical attributes of miniscule mass and/or momentum and high interfacial free energy [6,7], resulting in low flotation recoveries and rates. Two approaches can be used to improve the probability of bubble–particle collision [8]: one is to produce tiny bubbles suitable for capturing fine particles through electrolytic water (electrolytic flotation or electroflotation) [9,10] and hydraulic cavitation [11]. The other is to induce fine particle aggregation [12]. Fine particles can be aggregated by using electrolytes [13], polymer flocculants [14], nonpolar oils [15], microbubbles [16] or surfactants [17]. The aggregation mechanism varies depending on the added agent [18].

The aggregation of fine particles after their surfaces are rendered hydrophobic by the adsorption of a surfactant is known as hydrophobic flocculation [19]. Warren developed the term ‘shear flocculation’ to refer to the aggregation of ultrafine scheelite under high-shear agitation after being rendered hydrophobic by sodium oleate (NaOL, C_17_H_33_CO_2_Na) [20,21]. Warren considered that the kinetic energy provided by agitation could overcome the energy barriers as suggested by DLVO theory. However, Yoon and Luttrell [22] stated that kinetic energy is insufficient to break through the energy barriers and that the energy is provided by hydrophobic interactions. Thus, shear flocculation is identical to hydrophobic flocculation, and stirring is performed to provide the hydrodynamic conditions necessary for particle collision. Hydrophobic flocculation has been documented by numerous studies, including works on the hydrophobic flocculation of hematite with NaOL [23], dodecyl amine acetate and Aero 801 (a mixture of petroleum sulphonate and mineral oil) [24]; scheelite with NaOL [20,21] and cassiterite with NaOL [25], sodium lauryl sulphate (SLS, C_12_H_25_SO₄Na) and S3903 [17]. The abovementioned studies demonstrated that the variables that influence hydrophobic flocculation include pH, collector concentration, stirrer speed, collector type and agitation time. The hydrophobic flocculation is dominated by the hydrophobic interactions amongst particles [26]. Hydrocarbon chain association and hydrophobic interaction induce particle aggregation [23]. Thus, the carbon chain lengths of collectors affect the efficacy of hydrophobic flocculation. Numerous studies have demonstrated that collectors with long chain lengths can induce the formation of large mineral flocs [23]. Fine scheelite and cassiterite could form hydrophobic flocs in the presence of NaOL but not in the presence of benzohydroxamic acid (BHA, RCONHOH) [25]. The hydrophobic flocculation of fine cassiterite and tourmaline could not be induced by styrene phosphonic acid (SPA, C_8_H_9_O_3_P) but could be induced by NaOL, sodium lauryl sulphate (SLS, C_12_H_25_SO₄Na) and the Cyanamid reagent S3903, which contains a sodium salt of a long-chain alkyl derivative of aspartic acid as an active ingredient [17]. BHA and SPA are collectors with short hydrocarbon chains that impart minerals with a weak hydrophobicity. By contrast, NaOL, SLS and the Cyanamid reagent S3903 are collectors with long hydrocarbon chains and can render mineral surfaces strongly hydrophobic. Thus, the carbon chain length of a collector evidently plays a crucial role in hydrophobic flocculation. However, previous studies mainly focused on reagents with varying carbon chain lengths and functional groups. The effects of reagents with the same functional groups but various carbon chain lengths on hydrophobic flocculation have been rarely studied. Therefore, further research is warranted.

Hydroxamic acids, especially salicyl [27,28], benzo [29,30] and alkyl [31,32,33] hydroxamic acids, are frequently used as collectors in cassiterite flotation. When salicyl and benzo hydroxamic acids are used individually, metal ions are typically used as activators to enhance the cassiterite recovery [27,29,34,35,36]. Alkyl hydroxamic acids can be used individually in cassiterite flotation. The abilities of alkyl hydroxamic acids to collect cassiterite [31,32,33] and the surface activity of hydroxamate increase with the increase in carbon chain length. Therefore, compared with other collectors, hydroxamate, which has a longer carbon chain, can confer a stronger hydrophobicity to the cassiterite surface. Carboxylic acids and their derivatives, especially NaOL, are used as cassiterite collectors but have poor selectivity. Thus, this work selected hydroxamic acids [37,38]. By using alkyl hydroxamic acids with various carbon chain lengths as the collectors, this study investigated the influence of the collector’s carbon chain length on the hydrophobic flocculation of fine cassiterite. This work will provide a guideline for selecting reagents with appropriate carbon chain lengths to induce fine minerals to form hydrophobic flocs.

## 2. Results

### 2.1. The Effect of Alkyl Hydroxamic Acids with Various Carbon Chain Lengths on the Microflotation of Cassiterite

**Figure 1:**
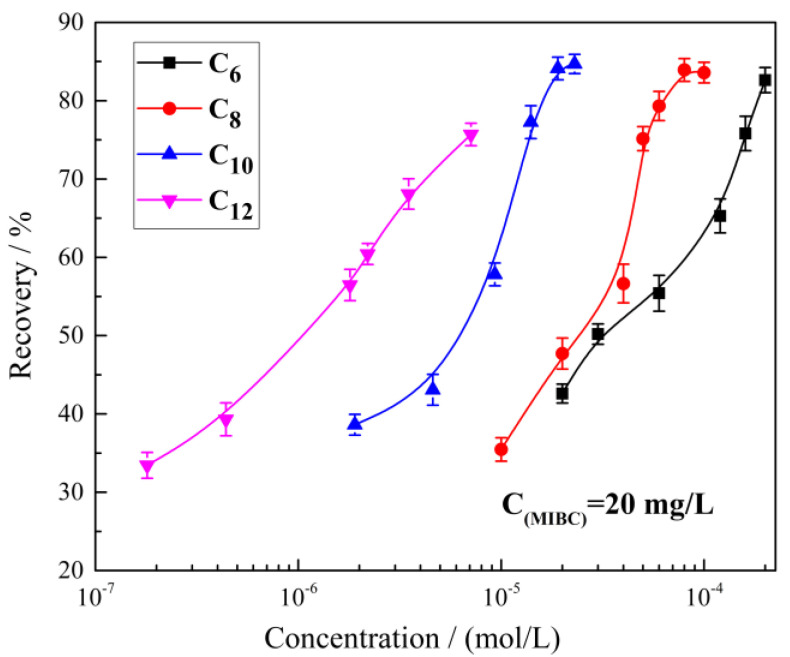
Cassiterite recovery as a function of the alkyl hydroxamic concentration (pH = 8.5).

The effect of the concentrations of the four alkyl hydroxamic acids on cassiterite floatability is shown in Figure 1. The hydroxamic acid concentration required to float cassiterite decreased with the increase in carbon chain length, indicating that hydroxamates with long carbon chains enhanced the cassiterite floatability. The flotation recoveries were between 30% and 85% because some cassiterite particles entered the concentrate through foam entrainment. The recovery could reach 30% even if only the frother was added. Some cassiterite particles were too fine to collide with and adhere to bubbles. Thus, this fraction of cassiterite could not be recovered even if the collector concentration was increased.

**Figure 2:**
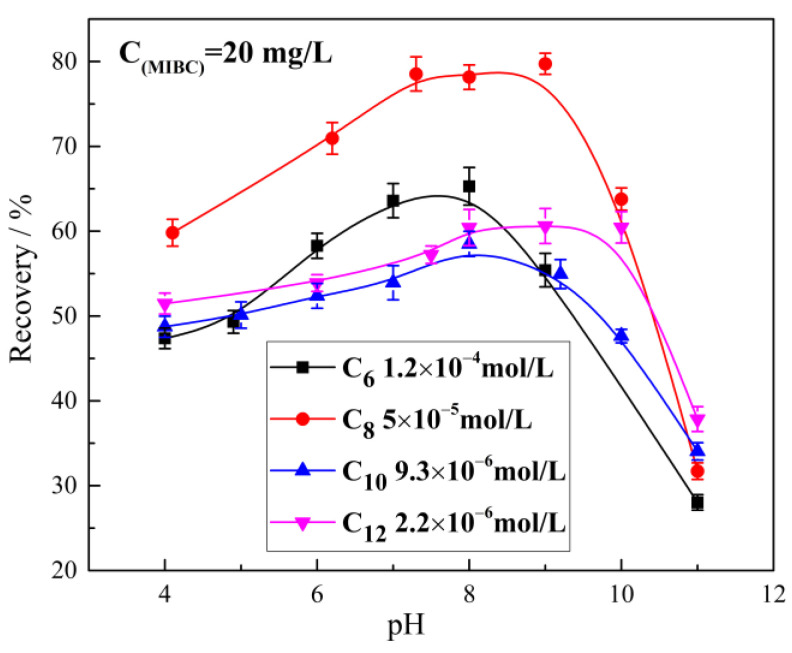
Cassiterite recovery as a function of pH.

The effect of pH on cassiterite floatability when different hydroxamates were used as collectors is presented in Figure 2. The cassiterite showed the best floatability under weakly alkaline conditions (pH 7–9). Under acidic conditions, its floatability decreased slowly with the decrease in pH. Under strongly alkaline conditions (pH > 9), its floatability decreased rapidly with the increase in pH.

### 2.2. The Effect of Alkyl Hydroxamic Acids with Various Carbon Chain Lengths on the Hydrophobic Flocculation of Cassiterite

#### 2.2.1. The Effect of Hexyl Hydroxamate on the Hydrophobic Flocculation of Cassiterite

**Figure 3:**
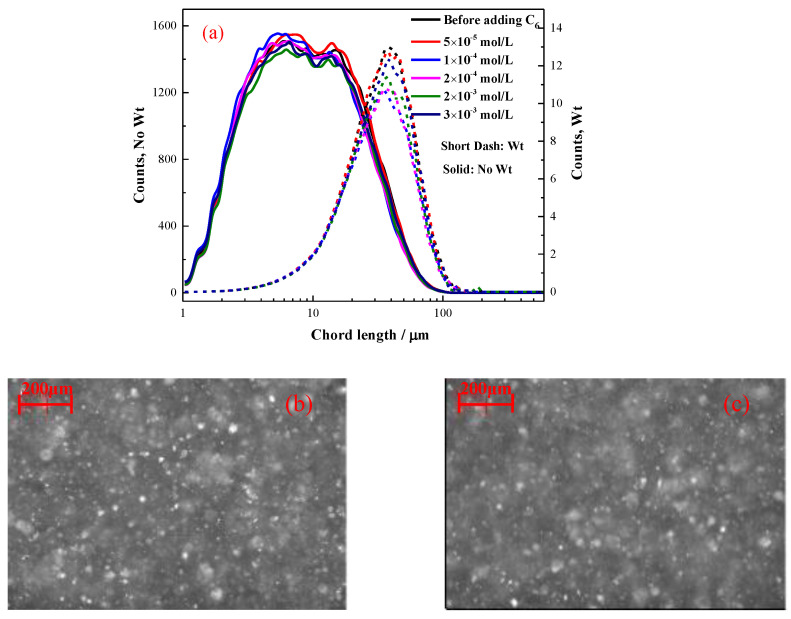
Aggregation of cassiterite by C_6_ at different concentrations and pH 8.5–9.0. (**a**) No-weighted and square-weighted CLDs of cassiterite suspensions before and after the addition of different concentrations of C_6_ at 20:00. (**b**,**c**) PVM images of cassiterite before and after the addition of 2 × 10^−3^ mol/L C_6_ at 20:00.

Figure 3 illustrates the flocculation of the cassiterite sample when 5 × 10^−5^, 1 × 10^−4^, 2 × 10^−4^, 2 × 10^−3^ or 3 × 10^−3^ mol/L C_6_ was used as the collector. The CLDs of the cassiterite particles almost remained unaltered at the time point of 20:00 after the addition of various concentrations of C_6_, suggesting that hydrophobic flocs did not form after the C_6_ addition. The PVM images did not show the presence of any significant aggregations, further indicating that flocs did not form after the addition of C_6_.

#### 2.2.2. The Effect of Octyl Hydroxamate on the Hydrophobic Flocculation of Cassiterite

**Figure 4:**
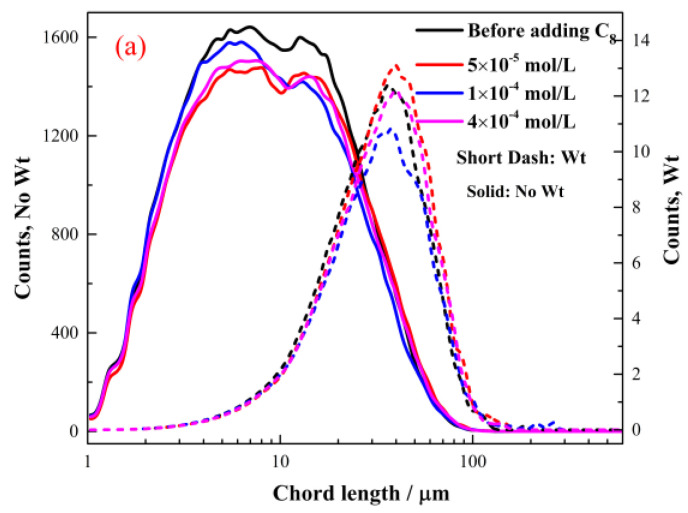

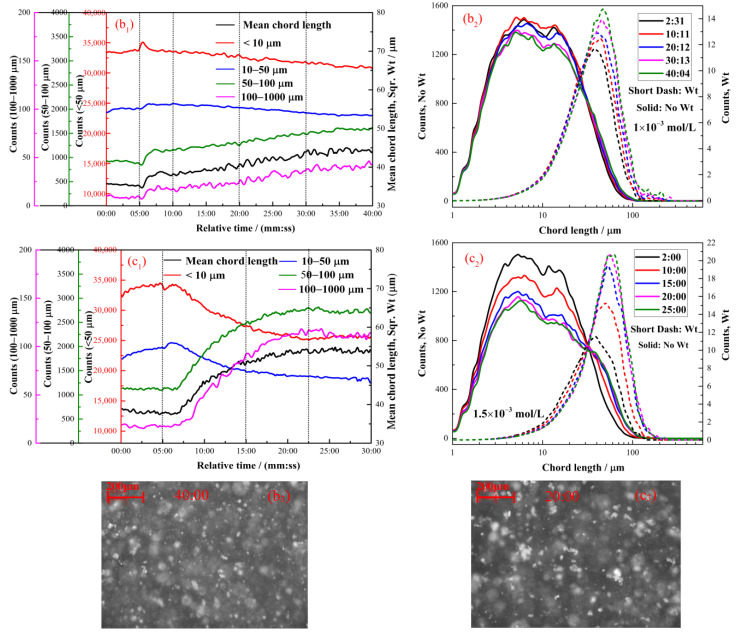
Aggregation of cassiterite by different C_8_ concentrations at pH 8.5–9.0. (**a**,**b_2_**,**c_2_**) No-weighted and square-weighted CLDs of the cassiterite suspension before and after the addition of different concentrations of C_8_ at different times. (**b_1_**,**c_1_**) Counts and square-weighted mean chord length of the cassiterite suspension as a function of time after the addition of 1 × 10^−3^ and 1.5 ×10^−3^ mol/L C_8_. (**b_3_**,**c_3_**) PVM images of cassiterite before and after the addition of 1 × 10^−3^ and 1.5 × 10^−3^ mol/L C_6_ at 40:00 and 20:00, respectively.

Figure 4 depicts the flocculation of the cassiterite sample when 5 × 10^−5^, 1 × 10^−4^, 4 × 10^−4^, 1 × 10^−3^ or 1.5 × 10^−3^ mol/L C_8_ was used as the collector. The CLDs shown in Figure 4a remained unchanged at 20:00 after the addition of 5 × 10^−5^, 1 × 10^−4^ and 4 × 10^−4^ mol/L C_8_, indicating that hydrophobic flocs did not form. Figure 4(b_1_) shows that after the addition of 1 × 10^−3^ mol/L C_8_, the mean chord length and coarse particle counts (50–100 and 100–1000 μm) gradually increased with stirring time, whereas the fine particle counts (−10 and 10–50 μm) decreased. The peak of the square-weighted CLD in Figure 4(b_2_) shifted from 38 μm at 02:31 to 44 μm at 40:04. This finding indicated that the fine cassiterite particles aggregated slowly and that the aggregates were very small. Furthermore, no noticeable aggregates were found in the PVM image captured at 40:00 and shown in Figure 4(b_3_).

Figure 4(c_1_) illustrates that the changes in the mean chord length and particle counts caused by the addition of 1.5 × 10^−3^ mol/L C_8_ were similar to those induced by the addition of 1 × 10^−3^ mol/L C_8_. The changes occurred more rapidly when C_8_ was added at the concentration of 1.5 × 10^−3^ mol/L than at other concentrations. At approximately 20:00, the system tended to reach equilibrium, and no change occurred. The peak of the square-weighted CLD shown in Figure 4(c_2_) shifted from 38 μm at 02:00 to 58 μm at 25:00. Small aggregates were observed in the PVM image captured at 20:00 and presented in Figure 4(c_3_). The fine cassiterite particles aggregated to form small flocs.

#### 2.2.3. The Effect of Decyl Hydroxamate on the Hydrophobic Flocculation of Cassiterite

**Figure 5:**
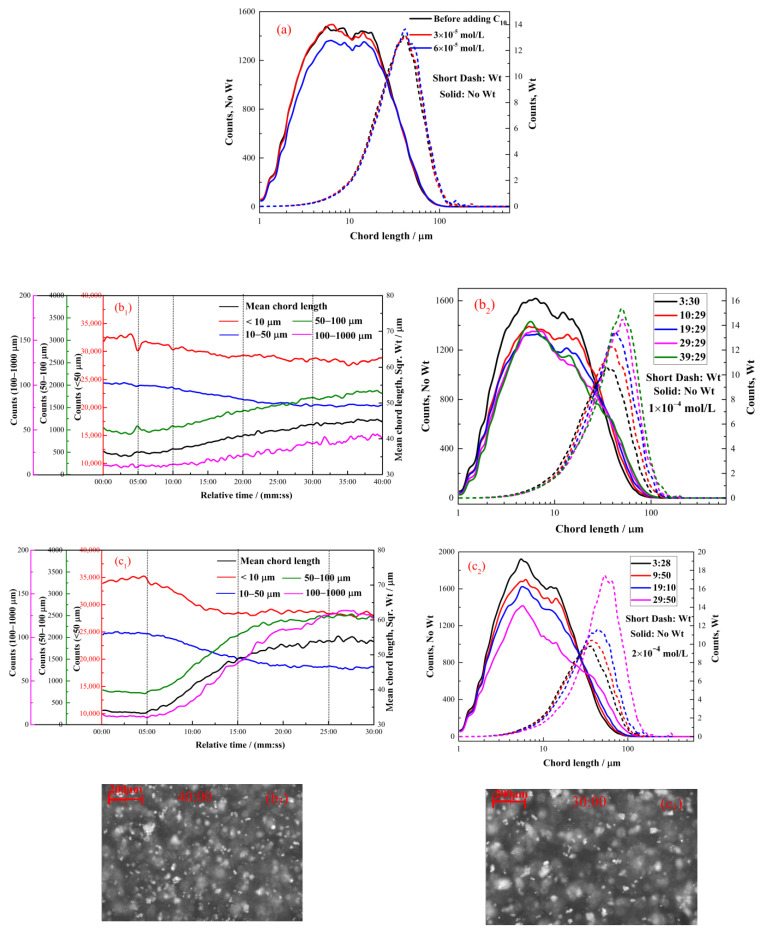
Aggregation of cassiterite by different C_10_ concentrations at pH 8.5–9.0. (**a**,**b_2_**,**c_2_**) No-weighted and square-weighted CLDs of the cassiterite suspension before and after the addition of different concentrations of C_10_ at different times. (**b_1_**,**c_1_**) Counts and square-weighted mean chord length of the cassiterite suspension as a function of time after adding 1 × 10^−4^ and 2 × 10^−4^ mol/L of C_10_. (**b_3_**,**c_3_**) PVM images of cassiterite before and after the addition of 1 × 10^−4^ and 2 ×10^−4^ mol/L C_10_ at 40:00 and 30:00, respectively.

Figure 5 illustrates the flocculation of the cassiterite sample with 3 × 10^−5^, 6 × 10^−5^, 1 × 10^−4^ or 2 × 10^−4^ mol/L C_10_ as the collector. Figure 5a shows that hydrophobic flocs did not form with the addition of 3 × 10^−5^ and 6 × 10^−4^ mol/L C_10_. As depicted in Figure 5(b_1_), the addition of 1 × 10^−4^ mol/L C_10_ caused the gradual aggregation of fine cassiterite particles. The peak of square-weighted CLD in Figure 5(b_2_) shifted from 37 μm at 03:30 to 49 μm at 39:29, indicating that the aggregates were very small in size. Small aggregates were observed in the PVM image provided in Figure 5(b_3_).

Figure 5(c_1_) presents the changes in the mean chord length and particle counts when 2 × 10^−4^ mol/L C_8_ was used as the collector. At approximately 20:00, the system tended to reach equilibrium, and changes barely occurred. Figure 5(c_2_) indicates that the peak of the square-weighted counts of CLD shifted from 36 μm at 03:28 to 57 μm at 29:50. Aggregates in the PVM image in Figure 5(c_3_) were larger than those in Figure 5(c_2_). This finding indicated that the fine cassiterite particles formed flocs.

#### 2.2.4. The Effect of Dodecyl Hydroxamate on the Hydrophobic Flocculation of Cassiterite

**Figure 6:**
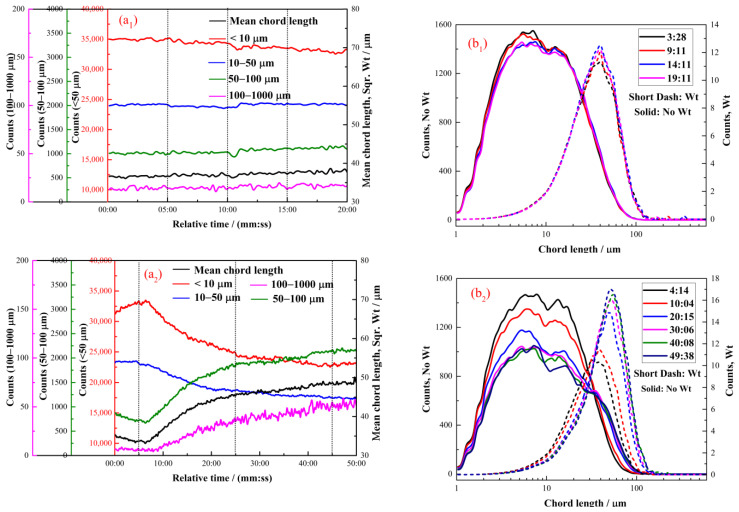

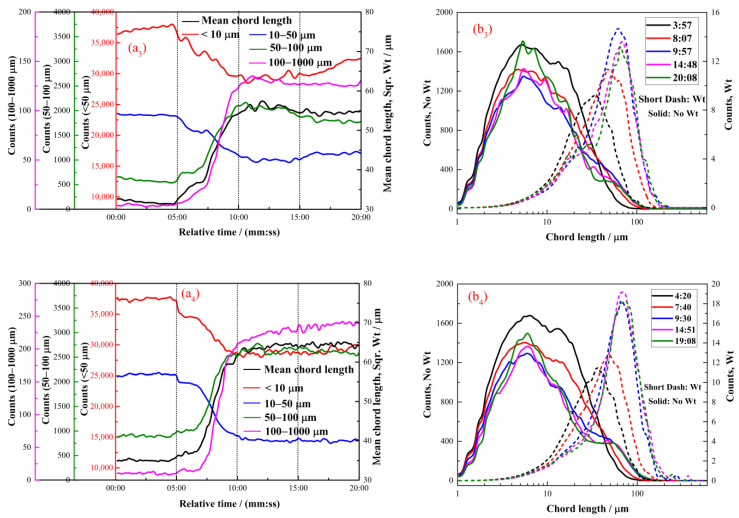
Aggregation of cassiterite by different C_12_ concentrations at pH 8.5–9.0. (**a**) Counts and square-weighted mean chord length of the cassiterite suspension as a function of time. (**b**) No-weighted and square-weighted CLDs of the cassiterite suspension at different times. (C = 1 × 10^−5^ mol/L—(**a_1_**,**b_1_**), C = 2 × 10^−5^ mol/L—(**a_2_**,**b_2_**), C = 4 × 10^−5^ mol/L—(**a_3_**,**b_3_**), C = 8 × 10^−5^ mol/L—(**a_4_**,**b_4_**).).

**Figure 7:**
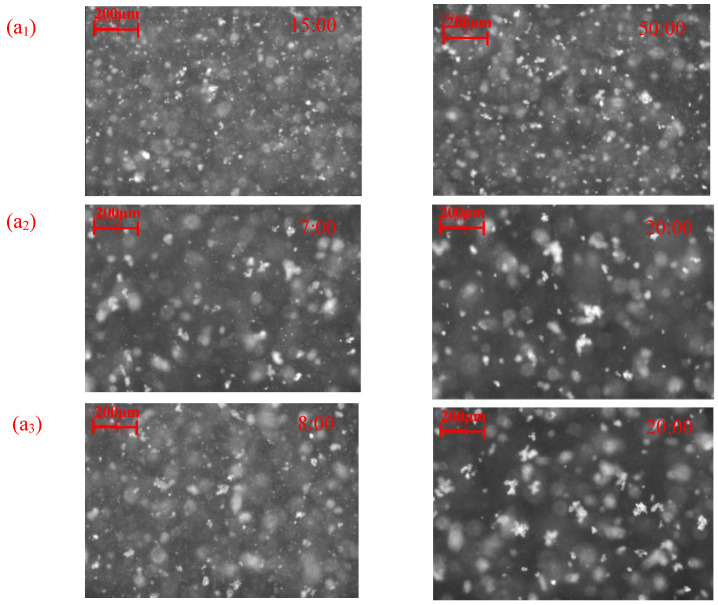
PVM images of cassiterite in the presence of different C_12_ concentrations at different times. (C = 2 × 10^−5^ mol/L—(**a_1_**), C = 4 × 10^−5^ mol/L—(**a_2_**), C = 8 × 10^−5^ mol/L—(**a_3_**).).

Figure 6 illustrates the flocculation of the cassiterite sample when 1 × 10^−5^, 2 × 10^−5^, 4 × 10^−5^ or 8 × 10^−5^ mol/L dodecyl hydroxamate (C_12_) was used as the collector. The PVM images of flocs are displayed in Figure 7. Figure 6(a_1_,b_1)_ illustrate that hydrophobic flocs did not form after the addition of 1 × 10^−5^ mol/L C_12_. Figure 6(a_2_,b_2_) indicate that the addition of 2 × 10^−5^ mol/L C_12_ caused particles to aggregate slowly. Figure 6(b_2_) shows that the peak of the square-weighted counts of CLD shifted from 35 μm at 04:14 to 53 μm at 49:38, indicating that the aggregates were small. Small aggregates were observed at 50:00 in the PVM image provided in Figure 7(c_1_).

Figure 6(a_3_,b_3_,a_4_,b_4)_ illustrate that the changes in aggregation status in the presence of 2 × 10^−5^ and 4 × 10^−5^ mol/L C_8_ were similar. At approximately 15:00, the systems tended to equilibrate, and no changes occurred. Figure 6(a_3_,a_4_) demonstrate that the peaks of square-weighted CLDs shifted from 34 μm at 03:57 to 65 μm at 20:08 and from 36 μm at 04:20 to 72 μm at 19:08, respectively. The PVM images in Figure 7(a_2_,a_3_) indicate that large aggregates approximately 50 μm in size were observed at approximately 20:00. Figure 7(a_1_–a_3_) clearly show that the aggregates enlarged with the increase in stirring time and C_12_ concentration.

### 2.3. The Interaction Energy Estimation by Extended DLVO Theory

**Figure 8:**
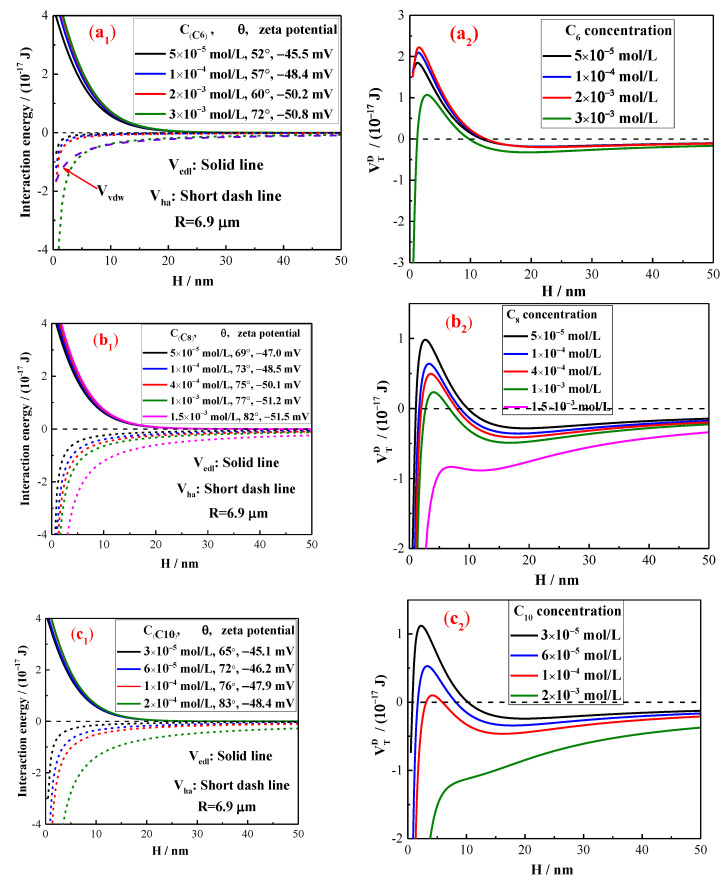

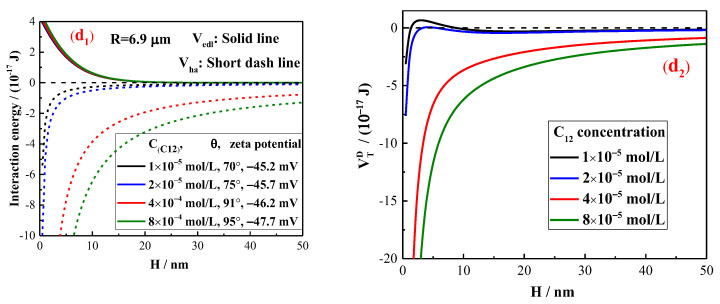
Diagram of the extended DLVO interaction energy as a function of the separation distance between cassiterite particles in the presence of alkyl hydroxamic acids. (C_6_—(**a_1_**,**a_2_**), C_8_—(**b_1_**,**b_2_**), C_10_—(**c_1_**,**c_2_**), C_12_—(**d_1_**,**d_2_**)).

The interaction energies between the cassiterite particles in the presence of alkyl hydroxamic acids with various carbon chain lengths are shown in Figure 8. These results were estimated in accordance with the extended DLVO theory on the basis of Equations (2)–(8). The radius value corresponding to D_50_ was used in the calculation. Only the curve of the van der Waals interaction energy is presented in Figure 8(a_1_). The data in Figure 8(a_1_–d_1_) show that the absolute value of the zeta potentials and contact angles of cassiterite increased with the increase in the concentrations of the hydroxamates. The energies of electrostatic and hydrophobic interactions amongst particles increased. However, the increments in electrostatic interaction energy were smaller than those in hydrophobic interaction energy. In some hydrophobic flocculation systems, the van der Waals and electrostatic interaction energies between the hydrophobic particles are one or two orders of magnitude smaller than those of the hydrophobic interaction energy [26]. Cassiterite did not aggregate in the presence of various concentrations of C_6_ because the existence of high energy barriers amongst particles resulted in repulsive interactions amongst particles when they were close to each other (Figure 8(a_2_)). When C_8_, C_10_ and C_12_ were used as collectors, the energy barrier amongst particles decreased with the increase in hydroxamate concentration (Figure 8(b_2_–d_2_)). The energy barrier of the total interaction energy could disappear when the collector concentration reached a particular level. The energy barrier amongst particles can be broken when it is less than the kinetic energy provided by agitation [22]. When the energy barrier is broken by agitation or disappears, aggregates can form amongst particles. The results of the FBRM experiment show that the lowest concentrations of C_8_, C_10_ and C_12_ that could induce cassiterite aggregation were approximately 1 × 10^−3^, 1 × 10^−4^ and 2 × 10^−5^ mol/L, respectively, indicating that agitation could overcome the energy barriers of the total interaction energies in the presence of 1 × 10^−3^, 1 × 10^−4^ and 2 × 10^−5^ mol/L C_8_, C_10_ and C_12_, respectively, shown in Figure 8(b_2_–d_2_). When the concentrations of the collectors were further increased, the energy barrier between particles disappeared, and the particles could aggregate.

## 3. Materials and Methods

### 3.1. Single Cassiterite Sample and Reagents

The cassiterite sample used in this work is identical to that in [33], but the particle size was processed to be finer for this investigation. The D_10_, D_50_, D_90_ and vol. weighted mean particle sizes were 2.08, 13.75, 36.04 and 16.72 μm, respectively, which were obtained by a laser-based particle size analysis instrument (Mastersizer2000, Malvern Instruments, Malvern, Worcestershire, UK).

The alkyl hydroxamates utilised in this work are the same as these in [33] and are hexyl hydroxamate (C_6_), octyl hydroxamate (C_8_), decyl hydroxamate (C_10_) and dodecyl hydroxamate (C_12_). As pH regulators, hydrochloric acid (HCl) and sodium hydroxide (NaOH) were obtained from Sinopharm Chemical Reagent Co., Ltd., Shanghai, China. Methyl isobutyl carbinol (MIBC, analytically pure) was used as the frother and purchased from Aladdin Biochemical Technology Co., Ltd., Shanghai, China. Deionised water was used throughout the experiments.

### 3.2. Microflotation Tests

Microflotation tests were performed using an inflatable hanging slot flotation apparatus (XFGC II) with a set impeller speed of 1900 r/min. In a 40 mL cell, 2.0 g of cassiterite sample was used for each test. HCl or NaOH was first added to adjust the solution pH and conditioned for 3 min. Then a necessary concentration of collector was added and stirred for 3 min. Finally, MIBC was added and stirred for 1 min. The flotation was performed for a total of 3 min.

### 3.3. Focused Beam Reflectance Measurement and Particle Video Microscope Observation

Flocculation processes were monitored by using a ParticleTrack G400 focused beam reflectance measurement (FBRM) probe (Mettler Toledo, Columbus, OH, USA), which can detect particles between 0.5 and 2000 μm in size. During the measurement, the probe was submerged in the suspension and a focused laser beam was rotated at the rate of 2 m/s to scan the particle that passed through the sapphire window. The chord length of the particle was calculated by multiplying its scanning time by its speed. Thousands of chord length data were detected per second to produce the chord length distribution (CLD). The chord length increases when flocculation occurs [39]. The distribution of the measured chord lengths of particles is a sensitive indicator that may represent the distribution of particle size [40]. A ParticleView V19 particle video microscope (PVM, Mettler Toledo, USA) with a resolution greater than 2 μm was used to view the structures of particles and flocs in situ. FBRM and PVM were conducted synchronously to obtain the particle size distributions and images. During measurement, chord length data were collected every 10 s and PVM images were collected every 20 s. iC FBRM^TM^ software was used to collect and analyse the FBRM data.

The influence of the hydroxamate concentration on the cassiterite flocculation was investigated on the basis of FBRM particle size analysis and PVM observations. Real-time recordings of the chord length count, CLD and square-weighted mean chord length of cassiterite particles were taken by conducting FBRM. PVM was used simultaneously to acquire the particle images. No-weight CLD provides enhanced number-sensitive information on fine particle counts, whereas the square-weighted CLD provides volume-sensitive information on aggregated coarse particles [40].

Before a measurement, 3.0 g of a cassiterite sample was added into a 500 mL glass beaker then added with a certain amount of deionised water. The slurry was fully dispersed by ultrasonication for 5 min. For the measurement, the slurry was stirred by using a magnetic stirrer at 400 r/min. The FBRM system and PVM were started for data collection when the pH of the slurry had been adjusted to 8.5–9.0. The required amount of collector was added after 5 min of conditioning. The total volume of deionised water and added collector was 300 mL.

### 3.4. Zeta Potential and Contact Angle Measurements

The zeta potentials of cassiterite were measured as described in the literature [33]. The contact angles of cassiterite were measured by using a JY-82C video-based contact angle measuring device. High-grade cassiterite lump samples were inlaid with mould epoxy resin and polished by using silicon carbide papers and a 1, 0.5 and 0.25 μm diamond paste with a semiautomatic polishing device. The polished samples were ultrasonically cleaned then used for contact angle measurement. The samples were first submerged into a solution containing the necessary amount of collector at pH 8.5 for 15 min [41]. Afterwards, the samples were gently rinsed with deionised water. Finally, the samples were air dried. A microsyringe was used to inject a drop of deionised water onto the surface of the cassiterite sample during measurement. After 30 s of equilibrium, a picture of the water drop was taken. The contact angle value was determined by analysing the picture. The average value was calculated after at least 8 measurements of each cassiterite sample at various locations on its surface.

## 4. Theoretical Background

DLVO theory, a classical theory of colloid chemistry, is utilised to explain the aggregation and dispersion phenomena of colloids and the interactions amongst particles [18]. The classical DLVO theory states that van der Waals interaction energy (V_vdw_) and electrostatic interaction energy (V_edl_) govern the stability of a colloidal dispersion system. It can be expressed as follows:

V_T_ = V_vdw_ + V_edl_, (1)

where V_T_ is the overall interaction energy. When V_edl_ is greater than V_vdw_, the colloid suspension is in a dispersed state. Otherwise, it is aggregated. However, Churaev and Derjaguin stated that DLVO theory is applicable only to colloidal systems with colloidal contact angles between 20° and 40° [42]. In hydrophobic flocculation flotation, the particles are rendered hydrophobic by the collectors and aggregate on the basis of the hydrophobic interaction energy (V_hy_) [19]. The extended DLVO theory should be adopted to determine the stability of these systems. Therefore, the total interaction energies of the colloid and particle system can be expressed as follows:

V_T_ = V_vdw_ + V_edl_ + V_hy_. (2)

Although the hydrophobic flocculation system of the cassiterite particles does not entirely correspond to a colloidal system, the extended DLVO theory has been successfully applied to study the interaction energy between various ore particles [13,43]. In this study, the extended DLVO theory was also used to investigate the aggregation behaviour of cassiterite particles.

The van der Waals interaction energy between two symmetric particles with radii R (m) that had adsorbed onto the surfaces of collectors can be computed as follows [43]:

(3) $$V_{\mathrm{vdw}}=-\frac{R}{12}\left[\frac{{\left(\sqrt{A_{22}}-\sqrt{A_{33}}\right)}^{2}}{H}+\frac{2\left(\sqrt{A_{22}}-\sqrt{A_{33}}\right)\left(\sqrt{A_{11}}-\sqrt{A_{22}}\right)}{H+\delta }+\frac{{\left(\sqrt{A_{11}}-\sqrt{A_{22}}\right)}^{2}}{H+2\delta }\right],$$

where A_11_, A_22_ and A_33_ are the Hamaker constants of the particle (2.56 × 10^−19^ J), the collector and the medium (water, 4.38 × 10^−20^ J), respectively. H is the separation distance between the two particle surfaces (nm) and δ is the thickness of the absorbed layer of a collector.

When calculating the electrostatic interaction energy, we assumed that the positions of the two colliding particles did not change and there was no slippage. For a symmetric system, the electrostatic interaction energy can be calculated as follows [13,43]:

(4) $$V_{\mathrm{edl}}{=2\pi R\varepsilon }_{0}\varepsilon_{r}\psi^{2}\mathrm{Ln}[1+\exp(-\kappa H)],$$

where ε_0_ is the absolute dielectric constant (8.854 × 10^−12^ F/m), ε_r_ is the dielectric constant of water (78.54), ψ is the surface potential of the particle (which can be approximately replaced by the zeta potential) and κ^−1^ is the Debye length, which can be calculated as [39]

(5) $$\kappa =\frac{\sqrt{C}}{0.304}.$$

The asymmetric hydrophobic interaction energy can be calculated as [44]

(6) $$V_{\mathrm{ha}}=\frac{-K_{132}}{6H}\frac{R_{1}R_{2}}{R_{1}{+R}_{2}},$$

where R_1_ and R_2_ are the radii of the two asymmetric particles. K is the hydrophobic force parameter, which can be compared directly with Hamaker constants and treated as the same form as Hamaker constants [45]:

(7) $$K_{132}=\sqrt{K_{131}K_{232}}.$$

The logarithms of the asymmetric hydrophobic force constants (K_131_ and K_232_) vary linearly with cosθ (θ is the contact angle of a particle). For a symmetric system, K_132_ can be acquired by applying the following empirical expression [22,40]:

(8) $${\mathrm{logK}}_{132}=-3.2\times \cos\theta -18.229.$$

## 5. Discussion

The amount of hydroxamate that had adsorbed on the cassiterite surface and the hydrophobicity of hydroxamate increase with the increase in the carbon chain length of hydroxamate [33]. Therefore, the concentrations of hydroxamic acids required to collect cassiterite declined with the increase in chain length (Figure 1 and Figure 2). However, given that some cassiterite particles entered the concentrate through foam entrainment and some particles were too fine to collide with and adhere to bubbles, the flotation recoveries of cassiterite were only between 30% and 85%, suggesting that a fraction of fine cassiterite could not be recovered through conventional flotation. Therefore, hydrophobic flocculation was used to aggregate fine cassiterite particles, increasing their susceptibility to a collision with bubbles.

The concentrations of hydroxamate required to aggregate the cassiterite particles decreased with the increase in carbon chain length (Figure 3, Figure 4, Figure 5, Figure 6 and Figure 7). The FBRM and PVM images show that fine cassiterite did not aggregate when C_6_, which has a shorter nonpolar carbon chain length than the other collectors, was utilised as the collector. When C_8_, C_10_ and C_12_ were used as the collectors, the lowest concentrations that could induce cassiterite particle aggregation decreased with the increase in hydroxamate concentrations. The extended DLVO theoretical calculation results demonstrate that this phenomenon is due to the low hydrophobic interaction energies imparted by C_6_ to cassiterite particles that resulted in high energy barriers amongst particles. The hydrophobic interaction energies conferred by the hydroxamate to the particles increased with the increase in the carbon chain length. When a surfactant with a long carbon chain is adsorbed onto a mineral surface, the hydrophobic attraction between the particles and the association of hydrocarbon chains cause aggregation [19]. Extended DLVO theory states that hydrophobic attraction is related to the contact angle of a particle. The addition of hydroxamic acids with long carbon chains could increase the contact angles of cassiterite. The strength of the hydrophobic associations is related to the carbon chain length of the surfactant. These findings indicate that the induction of hydrophobic aggregation by fine cassiterite was facilitated by the longer carbon chain of hydroxamic acid than that of the other collectors.

Figure 3, Figure 4, Figure 5, Figure 6 and Figure 7 indicate that the growth rate and apparent size of aggregates varied in the presence of various concentrations of a certain hydroxamate. For the quantitative demonstration of the aggregation growth rate (r, μm/min) of fine cassiterite particles when alkyl hydroxamic acids were used as collectors, the aggregation rates were calculated as

(9) $$r=\frac{d_{2}-d_{1}}{\;T_{2}-T_{1}},$$

where d_1_ and d_2_ (μm) are the square-weighted mean chord lengths of the particles at the relative time T_1_ and T_2_ (mm: ss), respectively. To increase the accuracy of the calculation, d1 was calculated by using the average of the square-weighted mean chord length of the particles from 00:00 to 05:00 and T_1_ = 05:00. d_2_ was used as the average of the square-weighted mean chord length of the particles in 1 min corresponding to T_2_. For example, when T_2_ = 20:00, d_2_ was used as the average of the square-weighted mean chord length between 19:00 and 20:00.

Table 1 presents the calculated results of r when alkyl hydroxamic acids at various concentrations were used as the collectors. In the calculation, T_2_ was selected in accordance with the time when the system reached equilibrium. The data in Table 1 show that r and the apparent floc size (d_2_) increased with the increase in hydroxamate concentration, indicating that the increase in hydroxamate concentration was conducive to the formation of hydrophobic flocs. Table 1 also illustrates that, compared with other collectors, the hydroxamic acid with a longer carbon chain could induce the cassiterite particles to form larger flocs at a lower concentration within a shorter time. This finding indicates that using a collector with a longer carbon chain facilitates floc formation by particles.

## 6. Conclusions

The effects of alkyl hydroxamic acids with various carbon chain lengths on the flotation and hydrophobic flocculation behaviours of fine cassiterite were studied by using flotation tests, FBRM and PVM. The interaction potential energies between the cassiterite particles were calculated by extended DLVO theory. The following conclusions were drawn:

(1) The hydroxamic acid concentration required to float cassiterite decreased with the increase in carbon chain length. The cassiterite flotation recoveries were between 30% and 85%.
(2) When C_6_ was used as a collector, the cassiterite particles could not form hydrophobic flocs. The lowest concentrations of C_8_, C_10_ and C_12_ required to induce the hydrophobic aggregation of cassiterite particles decreased with the increase in the carbon chain length. The lowest concentrations of C_8_, C_10_ and C_12_ were approximately 1 × 10^−3^, 1 × 10^−4^ and 2 × 10^−5^ mol/L, respectively.
(3) The aggregation growth rate and apparent floc size increased with the increase in the hydroxamic acid concentration. Compared with other collectors, the hydroxamic acid with a longer carbon chain could induce cassiterite particles to form larger flocs at a lower concentration within a shorter time.

## Figures and Tables

**Table 1:** Calculated results of aggregation growth rate.

Hydroxamic Acid	Concentration/(mol/L)	T_2_/min	d_2_ − d_1_/μm	r/(μm/min)
C_8_	1 × 10^−3^	40	44.2–35.6	0.25
	1.5 × 10^−3^	20	53.5–38.3	1.02
C_10_	1 × 10^−4^	40	45.1–35.7	0.27
	2 × 10^−4^	20	52.5–33.6	1.26
C_12_	2 × 10^−5^	40	47.4–34.2	0.38
	4 × 10^−5^	15	54.9–31.9	2.30
	8 × 10^−5^	15	64.3–31.5	2.92

## Data Availability

Not applicable.
